# Reduced neurovascular coupling is associated with increased cardiovascular risk without established cerebrovascular disease: A cross-sectional analysis in UK Biobank

**DOI:** 10.1177/0271678X241302172

**Published:** 2024-11-22

**Authors:** Sheng Yang, Alastair John Stewart Webb

**Affiliations:** 1 Wolfson Centre for Prevention of Stroke and Dementia, Nuffield, Department of Clinical Neurosciences, University of Oxford, Oxford, UK; 2 Department of Brain Sciences, 4615 Hammersmith Hospital, Imperial College London, London, UK

**Keywords:** Cerebral small vessel disease, endothelial function, hypertension, neurovascular coupling, vascular risk factors

## Abstract

Mid-life vascular risk factors predict late-life cerebrovascular diseases and poor global brain health. Although endothelial dysfunction is hypothesized to contribute to this process, evidence of impaired neurovascular function in early stages remains limited. In this cross-sectional study of 31,934 middle-aged individuals from UK Biobank without established cerebrovascular disease, the overall 10-year risk of cardiovascular events was associated with reduced neurovascular coupling (p < 2 × 10^−16^) during a visual task with functional MRI, including in participants with no clinically apparent brain injury on MRI. Diabetes, smoking, waist-hip ratio, and hypertension were each strongly associated with decreased neurovascular coupling with the strongest relationships for diabetes and smoking, whilst in older adults there was an inverted U-shaped relationship with DBP, peaking at 70–80 mmHg DBP. These findings indicate that mid-life vascular risk factors are associated with impaired cerebral endothelial-dependent neurovascular function in the absence of overt brain injury. Neurovascular dysfunction, measured by neurovascular coupling, may play a role in the development of late-life cerebrovascular disease, underscoring the need for further longitudinal studies to explore its potential as a mediator of long-term cerebrovascular risk.

## Introduction

Cerebral small vessel disease (CSVD) underlies 30% of ischaemic stroke, 80% of haemorrhagic stroke and up to 40% of dementia, and is strongly associated with mid-life vascular risk factors including hypertension, obesity, smoking and diabetes.^1–7^ These vascular risk factors are associated with cerebrovascular outcomes, including both large vessel stroke and small vessel disease, throughout the entire lifespan, and may precede overt disease by many decades.^8–11^ However, the short term effects of increased vascular risk factors on neurovascular function, prior to development of overt cerebrovascular disease, remain uncertain.

CSVD is associated with cerebrovascular endothelial dysfunction manifest as blood-brain barrier breakdown and decreased cerebrovascular reactivity,^12,13^ but it is unclear if endothelial dysfunction causes CSVD or is secondary to it. If causative, endothelial dysfunction should be present and associated with mid-life vascular risk factors before the development of overt CSVD on current neuroimaging markers. This would then identify a biomarker of early cerebrovascular injury and a potential treatment target for short-term titration of drugs for potential long-term clinical benefits.^14–16^ However, no methods for assessing cerebrovascular endothelial dysfunction have previously been available in large cohorts.

The vascular response to neuronal activity (neurovascular coupling, NVC) shares vasodilatory mechanisms with direct tests of endothelial function,^17–19^ and is associated with chronic cerebrovascular disease,^20,21^ thus providing a novel measure of cerebrovascular endothelial function applicable in large populations. In UK Biobank (UKB), over 35,000 participants underwent blood-flow sensitive blood-oxygen-level-dependent functional magnetic resonance imaging (BOLD-fMRI) during a visual task. This was initially designed to assess regional brain responses to emotive faces, resulting in minimal results.^22,23^ However, the absolute blood flow response to images presented during this task also provides a measure of the vasodilatory neurovascular response.

We therefore tested whether this readily-available index of neurovascular function in UKB is associated with increased vascular risk in subjects with no history of cerebrovascular disease and without clinically apparent cerebrovascular disease on MRI.

## Material and methods

### Participants

UK Biobank is an ongoing large-scale cohort study that collects data on demographics, lifestyle factors, clinical records, and imaging data from 502,540 middle-aged individuals residing in community settings, recruiting from 22 different centres across the UK.^24^ At face-to-face follow-up visits, 100,000 participants are planned to undergo an extensive MRI imaging assessment, included task-related fMRI data, of whom >50,000 participants have now been imaged, and processed data is available for the majority.^25^

This analysis includes participants with adequate quality, processed MRI imaging data, excluding participants with conditions that may directly affect NVC: multiple sclerosis or other demyelinating disorders, cerebral infarction, brain haemorrhage, encephalitis, brain abscesses, brain tumour, and systemic lupus erythematosus. These diagnoses were defined by self-reported stroke history and healthcare-linked data coded according to the International Statistical Classification of Diseases and Related Health Problems (ICD) version ten. Participants with unrealistic or unphysiological demographic and clinical variables were excluded, including systolic blood pressure (SBP) <80 or >220 mmHg and diastolic blood pressure (DBP) <40 or >120 mmHg, or if the SBP was equal to or lower than DBP.

This study was conducted with UKB data (https://www.ukbiobank.ac.uk/) under application number 41213. The UKB team obtained ethical approval from the North West Multi-Centre Research Ethical Committee, in accordance with the 1975 Declaration of Helsinki, to ensure ethical oversight, participant consent, and data collection. All participants provided written informed consent. UKB data is accessible to eligible researchers following a direct application.

### UK biobank variables

The brain imaging data were acquired on 3-Tesla MRI scanners, with methodological details available in prior publications^24–27^ and summarized in Table S1. All the imaging-derived phenotypes (IDPs) included in this paper are listed in Table S2. UKB data was retrieved on 29^th^ March 2023.

The primary outcome for this analysis, NVC, was estimated as the median BOLD signal change to a standardized simple shape, used as a control in the visual shapes-faces task, within a group-defined mask (Figure S1) predominantly including the visual cortex (i.e., shapes response versus baseline; IDP: 20249). This method of measuring BOLD signal change (by the Featquery function built in FSL^28^) was adopted in previous, smaller-scale studies.^29–31^ Sensitivity analyses excluded potential confounding variables including higher (upper one-third) head motion (IDP: 25742) and CSVD burden (IDP: 25781), represented by normalized white matter hyperintensity (WMH) relative to total brain volume.^32,33^

The independent variables in this analysis included age (years), age-squared (age^2^), sex, race, body mass index (BMI), waist-to-hip ratio (WHR), blood pressure, smoking, a reported history of hypertension, diabetes, hypercholesterolaemia, and use of insulin, blood pressure or cholesterol control medications. In UKB, these variables were derived from self-reported answers to a touchscreen questionnaire, including lifestyle and past and current medical conditions, and combined with the corresponding ICD diagnosis for diabetes, hypertension or hypercholesterolaemia from health-care linked data. Serum biochemical markers were ascertained from baseline sampling and analysis by the UKB study, as previously reported (Table S2), including fasting blood markers: glycated haemoglobin (HbA1c), total cholesterol, triglycerides, high-density lipoprotein cholesterol (HDL), and low-density lipoprotein cholesterol (LDL). All data were collected concurrently with NVC, except for serum markers. The blood biochemistry data was obtained during visits prior to the brain scans (mean time difference: 9.2 ± 2.1 years).

The overall 10-year risk of cardiovascular events was calculated by the atherosclerotic cardiovascular disease risk (ASCVD) from American College of Cardiology/American Heart Association,^34^ which estimates the risk of first occurrence of non-fatal myocardial infarction, congestive heart disease death, or fatal or nonfatal stroke as a function age, sex, race, smoking, diabetes, hypertension treatment, SBP, total cholesterol, and HDL, stratified into four risk groups: low (<5%), borderline (5–7.4%), intermediate (7.5–19.9%), and high (≥20%).^34^ Secondly, the SCORE2 estimate of 10-year risk of fatal and non-fatal cardiovascular disease in Europe was determined as a function of age, sex, region, smoking, diabetes, SBP, and non-HDL cholesterol.^35,36^

### Statistical analysis

Baseline demographics were presented as mean with standard deviation for continuous data and percentage for categorical data.

Categorical variables with up to 5% missing data (smoking, alcohol intake, hypertension diagnosis, diabetes diagnosis, and hypercholesterolemia diagnosis), which also included data coded as non-available, “prefer not to say,” or “do not know,” were conservatively assumed to be absent, thus set to “No” or “Never,” to avoid introducing assumptions or biases when dealing with the missing categorical data. For continuous variables with up to 5% missing data, multiple imputation by chained equations (MICE) was used to estimate missing values, by generating five imputed datasets with ten iterations each, performed separately for men and women.^37,38^ For both continuous and categorical variables with missing data exceeding 5%, imputation was not done but analyses were performed without these groups where possible.

Univariate associations between NVC and continuous variables were assessed by linear regression. Differences in NVC between binary groups were compared to the reference group using t-tests. Multivariable associations between NVC with age, sex, and vascular risk factors were assessed by general linear models (GLM), with and without adjustment for age and sex, or for age, sex and vascular risk factors. Risk factors included SBP, DBP, BMI, WHR, smoking status, hypertension, diabetes, and hypercholesterolaemia. Normality of continuous variables was assessed by Quantile-Quantile (Q-Q) plots, with log-transformation for non-normal variables. Continuous variables were scaled before inclusion in GLM models. Associations are presented as standardized beta coefficients (β) and p-values. A p-value of <0.05 was deemed significant, and statistical methods used were two-tailed.

Associations with blood pressure were performed both for SBP and DBP, and for steady state and pulsatile blood pressure as mean BP (MAP) and pulse pressure (PP).^39^ Analyses were performed both for concurrent blood pressure at the time of the MRI scan, and historic blood pressure at the time of recruitment to UKB. Sensitivity analysis was performed for participants with lower CSVD burden (lower 2/3^rd^ WMH volume), lower head motion during scanning (lower 2/3^rd^), normal-range blood pressure (SBP <120 and DBP <80 mmHg).

All analysis was performed in R with packages *mice* (data imputation), *RiskScorescvd* (ASCVD and SCORE2 calculation), and *ggplot2* (plotting).

## Results

### Study selection

38,556 of 42,764 participants undergoing a task-fMRI had adequate quality imaging with available NVC (median z-statistic) data. After excluding participants with confounding diagnoses (n = 1026) or extreme blood pressure values (n = 82), a total of 37,448 participants were included (Figure S2). Imputation was performed for missing SBP and DBP (1%), WHR (2%), and BMI (3%) data. Data required for calculation of 10-year cardiovascular event risk and blood biochemistry was available in 85.3% (n = 31,934) of participants. BMI and PP were non-normal due to a right-skew (Figure S3), and were log-transformed. Demographics of participants included at the time of the fMRI scan, as well as those included in the sensitivity analyses, are summarized in Table 1 and Tables S3, S4, S6, and S8.

**Table 1.** Baseline characteristics.

Variable	Whole population	Female	Male
Participants (n)	37448	19962 (53.3)	17486 (46.7)
NVC (z-statistic)	2.6 (1.4)	2.7 (1.4)	2.4 (1.3)
Age	64.0 (7.7)	63.4 (7.6)	64.6 (7.8)
White	34159 (91.2)	18150 (90.9)	16009 (91.6)
SBP (mmHg)	139.2 (18.6)	137.0 (19.5)	142.2 (17.2)
DBP (mmHg)	78.0 (10.4)	76.3 (10.4)	80.0 (10.1)
Weight (kg)	75.9 (15.0)	69.1 (13.0)	83.6 (13.2)
Height (cm)	169.0 (9.2)	162.8 (6.2)	176.0 (6.6)
BMI (kg/m^2^)	26.5 (4.4)	26.1 (4.7)	26.9 (3.9)
WHR	0.9 (0.1)	0.8 (0.1)	0.9 (0.1)
Current smoker	2279 (6.1)	1041 (5.2)	1238 (7.1)
Ex-smoker	12293 (32.8)	6103 (30.6%)	6190 (35.4)
Pack-year^Δ^	5.0 (11.5)	3.9 (9.6)	6.3 (13.2)
Hypertension	11206 (29.9)	4836 (24.2)	6370 (36.4)
Diabetes	2254 (6.0)	875 (4.4)	1379 (7.9)
Hypercholesterolaemia	3696 (9.9)	1377 (6.9)	2319 (13.3)
HbA1c (%)^†^	5.3 (2.6)	5.3 (2.6)	5.4 (2.6)
Total cholesterol (mg/dL)^†^	221.7 (41.7)	226.2 (41.3)	216.7 (41.5)
Triglycerides (mg/dL)^†^	145.0 (84.59)	124.9 (67.9)	167.9 (95.3)
HDL (mg/dL)^†^	57.2 (14.5)	63.2 (14.3)	50.5 (11.6)
LDL (mg/dL)^†^	138.6 (31.9)	138.9 (31.8)	138.2 (31.9)

^Δ^86.2%, 86.6%, 85.8% of participants had available smoking pack-year data. ^†^85.2%, 84.5%, 86.2% of participants had available blood chemistry data.

Values at the time of fMRI are presented, aside for baseline blood biochemistry measures which was taken on average 9.2 ± 2.1 years before fMRI. All variables, with the exception of baseline LDL (p = 0.68), were significantly different between male and female (p < 0.05). Continuous variables given as mean (sd) and frequencies as n (%). NVC: neurovascular coupling; SBP: systolic blood pressure; DBP: diastolic blood pressure; BMI: body mass index; WHR: waist-hip ratio; HbA1c: haemoglobin A1C; HDL: high-density lipoprotein; LDL: low-density lipoprotein.

**Figure 1.**
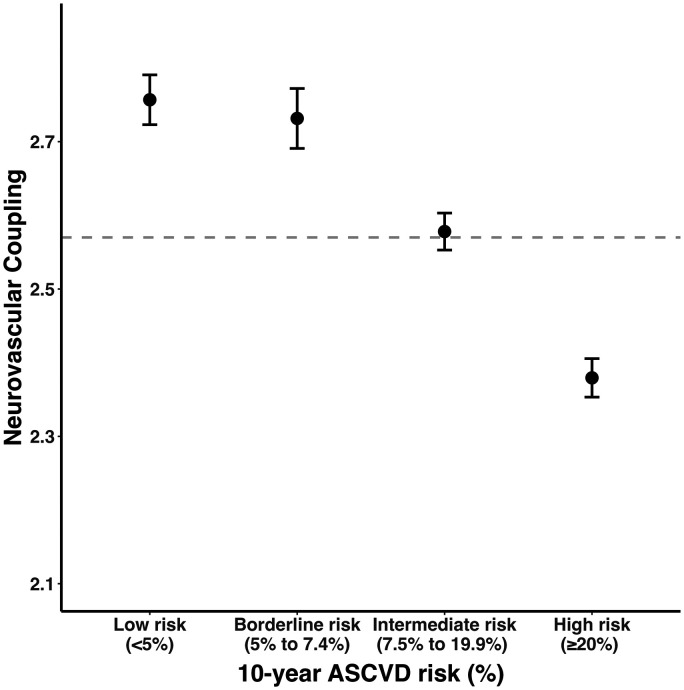
Neurovascular coupling decreases with increasing current 10-year cardiovascular disease risk. Neurovascular coupling is presented as dots (mean, expressed as z-statistic) with error bars (95% confidence interval). The size of each dot is proportional to the logarithm of the subgroup size. The horizontal dashed line marks the overall mean neurovascular coupling. ASCVD: atherosclerotic cardiovascular disease.

The overall 10-year risk of future cardiovascular events was strongly associated with reduced NVC, by both ASCVD (β = −0.116, p < 0.001) and SCORE2 (β = −0.103, p < 0.001), with a linear reduction in NVC across risk categories (Figure S4). Overall, there was a 13.8% reduction in standardized BOLD response between the highest-risk and lowest-risk ASCVD risk groups (Figure 1). There were similar associations in the sensitivity analysis, after excluding participants with a significant CSVD burden, with a 12.7% drop in response between highest and lowest risk group (Figure S4).

**Figure 2.**
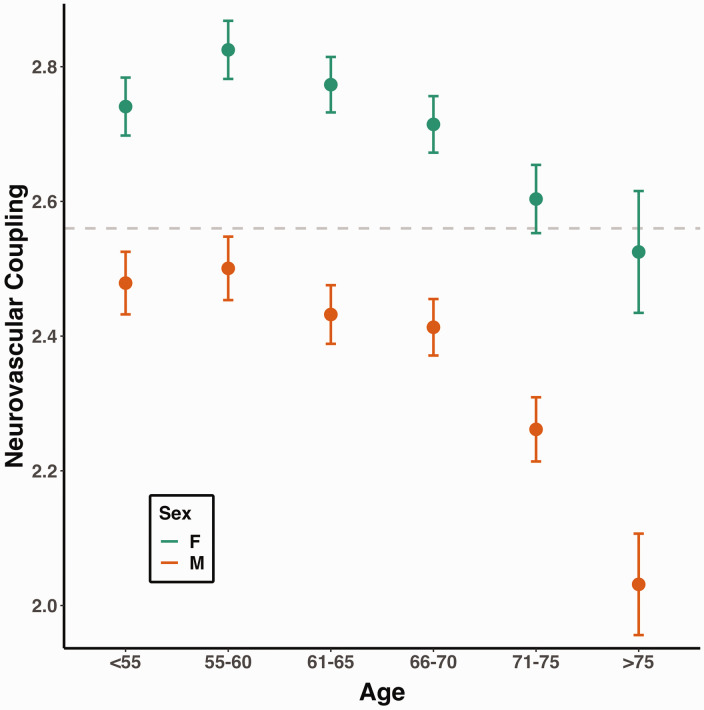
Neurovascular coupling is lower in male participants, and declines faster after middle-age. Neurovascular coupling is presented as dots (mean, expressed as z-statistic) with error bars (95% confidence interval). The size of each dot is proportional to the logarithm of the subgroup size. The horizontal dashed line marks the overall mean neurovascular coupling.

NVC was lower with increasing age and male sex. In both unadjusted and fully-adjusted models, NVC decreased by 7.2% and 5.6%, respectively (both p < 0.001) for each SD increment in age. The association between age and a lower NVC was stronger in participants aged 65 years or older, whilst males demonstrated lower mean NVC than females across both younger and older cohorts (Table 2, Figure 2).

**Table 2.** Association between neurovascular coupling with age, sex, and vascular risk factors.

Variable	Unadjusted		Age and sex adjusted		Fully adjusted	
	β	p-value	β	p-value	β	p-value
Age	−0.072	**<0.001**	–	–	−0.056	**<0.001**
Age^2^	−0.038	**<0.001**	–	–	−0.039	**<0.001**
Male sex	−0.250	**<0.001**	–	–	−0.240	**<0.001**
SBP	−0.038	**<0.001**	−0.001	0.79	−0.007	0.32
DBP	−0.005	0.34	0.014	**0.007**	0.016	**0.016**
BMI	−0.015	**0.003**	−0.003	0.53	−0.002	0.74
WHR^Δ^	−0.109	**<0.001**	−0.038	**<0.001**	−0.029	**<0.001**
Ex−smoker	−0.077	**<0.001**	−0.046	**<0.001**	−0.047	**<0.001**
Current smoker	−0.023	0.43	−0.020	0.48	−0.017	0.55
Hypertension^†^	−0.091	**<0.001**	−0.029	**0.011**	−0.026	**0.031**
Diabetes	−0.130	**<0.001**	−0.079	**<0.001**	−0.073	**0.001**
Hypercholesterolaemia	−0.087	**<0.001**	−0.009	0.60	−0.000	0.99

Associations are presented as standardized coefficients (β) and p-values from generalized linear models. The fully-adjusted model included: age, age^2^, sex, SBP, DBP, BMI, smoking, diabetes, hypercholesterolemia. ^Δ^BMI excluded. ^†^SBP and DBP excluded. SBP: systolic blood pressure; DBP: diastolic blood pressure; BMI: body mass index; WHR: waist-hip ratio.

In sensitivity analyses, there was a persistent association between increasing age and reduced NVC in participants without significant CSVD (unadjusted standardized β = −0.052, p < 0.001; adjusted model standardized β = −0.043, p < 0.001); and in participants in with limited head motion (unadjusted β = −0.020, p < 0.001; after adjustment β = −0.007, p = 0.38). Similarly, in normotensive participants, increasing age was associated with lower neurovascular coupling in the unadjusted model (β = −0.049, p = 0.001) and following adjustment for confounding variables (β = −0.051, p = 0.006; Table S5, S7, S9). Male sex was associated with lower NVC in all unadjusted and adjusted models (Table S5, S7, S9).

### Neurovascular coupling and blood pressure

A diagnosis of hypertension was associated with reduced NVC, with a 9.1% SD decrease in the unadjusted model (p < 0.001), a 2.6% SD decrease in fully-adjusted model (p = 0.031), and a consistent decrease in NVC across hypertensive stages (Table 2, Figure 4(a) and (b)). Among hypertensive subjects, those who reported taking blood pressure medication had significantly higher NVC compared to those who did not (p < 0.001).

**Figure 3.**
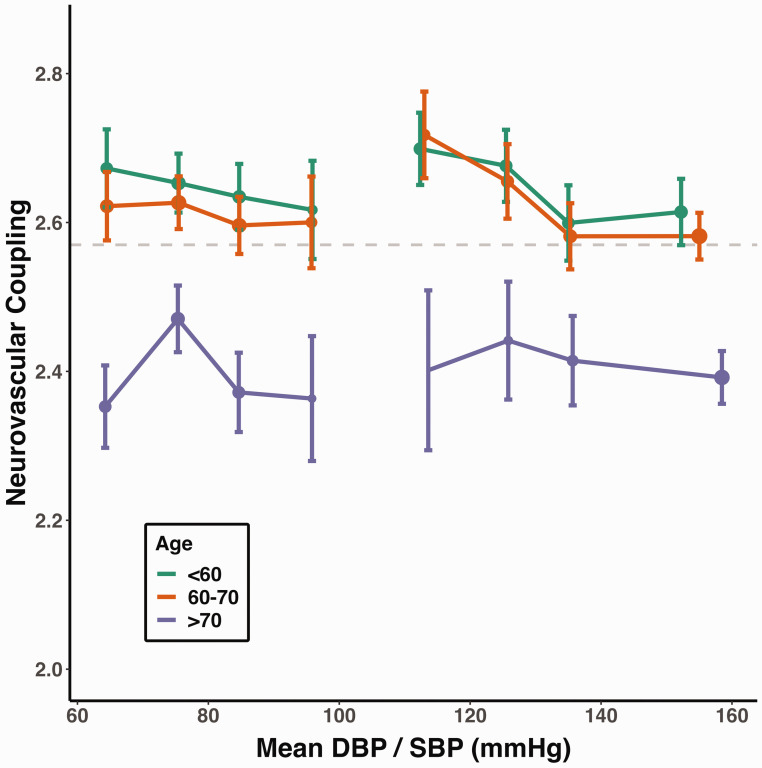
Higher blood pressure is associated with lower neurovascular coupling, while DBP in older participants shows an inverted U-shaped pattern. Neurovascular coupling is presented as dots (mean, expressed as z-statistic) with error bars (95% confidence interval). The size of each dot is proportional to the logarithm of the subgroup size. The horizontal dashed line marks the overall mean neurovascular coupling. Neurovascular coupling is stratified by age and blood pressure (DBP: <70, 70–80, 80–90, ≥90 mmHg; SBP: <120, 120–130, 130–140, ≥140 mmHg).

Similarly, NVC decreased with increasing SBP (Figure 3), which persisted with a reduced magnitude in both the age and sex-adjusted model and in the fully-adjusted model (Table 2). Conversely, although there was a non-significant relationship between increasing DBP and reduced NVC in an unadjusted model, this association became positive in both the age and sex and in the fully-adjusted model (Table 2), although the relationship was non-linear with the greatest NVC between 70–80 mmHg DBP in the older participant group (Figure 3).

Historic SBP and DBP showed the same pattern of associations with reduced NVC but the magnitude of the association was greater (Table S10). The pattern of association with blood pressure was similar in sensitivity analyses, with a stronger association in participants without evident CSVD or with normotension, and a similar magnitude of association in subjects without excessive head motion (Table S5, S7, S9).

Associations between steady state blood pressure (MAP) or log-transformed pulsatile pressure (PP) data demonstrated a 2.3% SD decrease in NVC for MAP and a 4.5% SD decrease for PP (both p < 0.001) in unadjusted models. However, in adjusted models increasing MAP was associated with greater NVC, whereas increasing PP remained associated with reduced NVC (Table S11).

### Neurovascular coupling and other vascular risk factors

Diabetes was the most strongly associated, reversible risk factor for reduced NVC in the fully adjusted model (Table 2). Type 2 diabetes demonstrated a stronger association with NVC reduction compared to type 1, although there were limited participants with type 1 diabetes (n = 149; Figure S5). These associations persisted but were slightly weaker in sensitivity analyses (Table S5, S7, S9). Among diabetic subjects, there was no significant difference in NVC between those taking and those not taking insulin (p = 0.55). When assessed by level of HbA1c, only an HbA1c of >8.0% was individually associated with reduced NVC, with non-significant reductions in NVC in participants with a modest increase in HbA1c (Figure S5), although there was a continuous association between HbA1c and reduced NVC (β = −0.037, p < 0.001, unadjusted).

**Figure 4.**
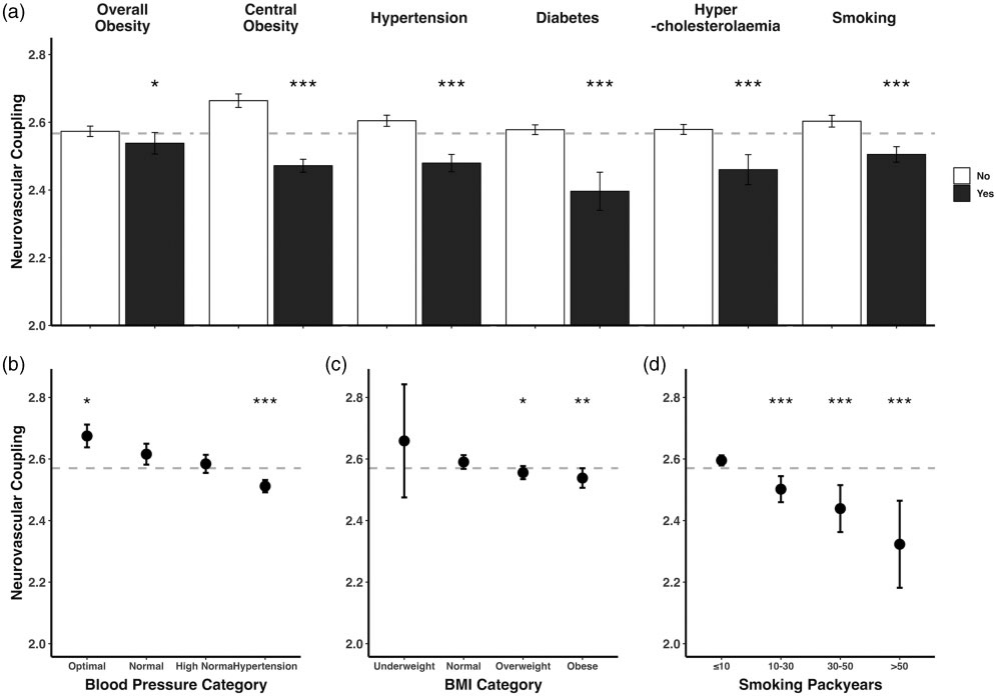
Decreased neurovascular coupling is associated with concurrent vascular risk factors. (a) Neurovascular coupling is presented as bars (mean, expressed as z-statistic) with error bars (95% confidence interval). The horizontal dashed line marks the overall mean neurovascular coupling. Overall obesity is defined at BMI ≥30, central obesity as waist-hip ratio ≤0.85 for female and ≤0.9 for male. Between-group comparisons by t-test (* unadjusted p < 0.05, ** unadjusted p < 0.01, *** unadjusted p < 0.001). (b–d) Neurovascular coupling is presented as dots (mean, expressed as z-statistic) with error bars (95% confidence interval). The size of each dot is proportional to the logarithm of the subgroup size. The horizontal dashed line marks the overall mean neurovascular coupling. Blood pressure is stratified to optimal (<120/80 mmHg), normal (120–129/80–84 mmHg, reference group), high normal (130–139/85–89 mmHg), and hypertension (≥140/90 mmHg). BMI is stratified to underweight (<18.5), normal (≥18.5 & <25, reference group), overweight (≥25 & <30), and obese (≥30). Between-group comparisons by ANOVA (unadjusted p < 0.05 for all variables) with post-hoc t-test (* unadjusted p < 0.05, ** unadjusted p < 0.01, *** unadjusted p < 0.001). Normal blood pressure, normal BMI, and ≤10 smoking packyears are the reference groups, respectively.

There was a consistent decrease in NVC with increasing log-transformed BMI, albeit to a somewhat lesser extent when accounting for other covariates. Conversely, central obesity estimated by WHR^40^ was more strongly associated with reduced NVC than BMI, with a particularly strong association after adjustment for age and sex, or age, sex and other vascular risk factors, such that increased WHR was more strongly associated with reduced NVC than blood pressure, hypertension, or hypercholesterolaemia in the fully adjusted model (Table 2, Figure 4(a) and (c)). These associations persisted but were weaker in sensitivity analyses (Table S5, S7, S9).

A diagnosis of dyslipidaemia was associated with a significant reduction in NVC, although this attenuated after adjustment for age, sex and vascular risk factors (Table 2, Figure 4(a)). Subjects with diagnosed dyslipidaemia taking lipid-lowering medications had significantly higher NVC compared to those who did not (p = 0.005). There was also an association between increased NVC and healthy triglycerides and high-density lipoprotein (HDL) levels at baseline, but no relationship between NVC and LDL and an increase in NVC in the highest quartile of total cholesterol (Figure S6).

A past history of smoking was strongly associated with reduced NVC. There was also a strong dose-response relationship between NVC and number of pack-years (Figure 4(a) and (d)), but there was no association with current smoking before (β = −0.023, p = 0.43), or after adjustment for age and sex (−0.020, p = 0.48) or all vascular risk factors (−0.017, p = 0.55) (Table 2).

## Discussion

In the UK Biobank population, an increased 10-year risk of cardiovascular events was associated with a reduced vasodilatory response to a standardized neuronal stimulus, consistent with impaired neurovascular coupling. NVC was reduced with each individual vascular risk factor, including hypertension, diabetes, a history of smoking and increased central obesity, with a dose response relationship for each factor and the strongest associations with diabetes and smoking. These relationships were consistent in participants with no clinically apparent CSVD, suggesting that cerebrovascular endothelial dysfunction occurs prior to development of CSVD, a prerequisite for causation. NVC therefore may be a new biomarker and treatment target.

The strong association between the predicted 10-year risk of cardiovascular events and impaired NVC suggests a potential influence of mid-life vascular risk factors on neurovascular dysfunction, before this is evident as symptomatic or structurally-evident disease, and may therefore mediates the development of late-life disease.^9,41,42^ In particular, the association with long-standing hypertension is consistent with associations between mid-life blood pressure with late-life ischaemic stroke;^43,44^ the stronger relationship between mid-life than late-life DBP with WMH;^8^ increased microstructural white matter damage before the development of WMH;^4^ and with late-life cognitive decline.^41,42^ In addition, the association between NVC and DBP reverses when modelled with SBP indicative of a protective effect of mean pressure in the context of highly pulsatile pressures, and consistent with positive associations between microstructural damage and mean BP when modelled with BP pulsatility.^4,45^ Furthermore, the inverted U-shaped relationship between DBP and NVC in older UKB participants matches the relationship reported between DBP and cognitive decline in older people.^41,46^ Similarly, the reduced NVC with diabetes, long-standing smoking and central obesity is consistent with their effects on endothelial injury^40,47–49^ and the associated risk of stroke,^3,50,51^ CSVD,^3,21,52–54^ and cognitive decline.^21,54–56^ The beneficial effects of medications that modify vascular risk factors suggest that targeting endothelial dysfunction via multiple different pathways may reduce progression of cardiovascular disease.

In this study female subjects demonstrated higher NVC than males. One potential factor is the vaso-protective role of sex hormones, particularly estrogen, with its vasodilatory effects through increased production of NO and reducing oxidative stress,^57–59^ and potential contributions in maintaining the structural integrity of the vascular endothelium and improving blood flow.^58,60^ Additionally, there are known differences in vascular risk profiles between males and females, with women developing vascular phenotypes associated with age and hypertension later in life.^61,62^ However, even after controlling for these factors in our models, a significant difference in NVC persisted between males and females, suggesting that sex alone may have an intrinsic effect on endothelial function independent of traditional risk factors.^63^ Future dedicated studies are needed to further elucidate the influence of sex on endothelial function and NVC.

The association between vascular risk factors and reduced NVC was only measured on BOLD-MRI in UKB. However, these associations are consistent with smaller cohorts measuring NVC by other modalities. Reduced NVC was associated with severity of hypertension on positron emission tomography (PET) scans in middle-aged, untreated hypertensive subjects^64^ and on transcranial Doppler (TCD) in hypertensive patients without CSVD,^65^ although there was no difference in response on TCD or near-infrared spectroscopy (NIRS) between controlled hypertension and normotensive controls.^66^ Similarly, NVC on TCD was reduced in smokers, even after smoking cessation^52,53^ and ‘default mode network (DMN)’ activity was reduced in diabetics compared to healthy controls.^67^ As these studies and ours were performed in largely healthy populations without established brain injury, and included modalities specific to arterial blood flow, the association with reduced NVC is consistent with a vascular rather than neuronal effect.

Impaired NVC, effects of vascular risk factors, and cerebrovascular injury are linked by endothelial dysfunction. Vascular risk factors such as chronic hypertension, smoking and diabetes directly cause endothelial dysfunction through induced oxidative stress, impaired vasodilation through decreased nitric oxide (NO), prostaglandin E2 (PGE2), and epoxyeicosatrienoic acids production, blood-brain barrier breakdown and activation of the inflammatory cascade,^17,68–71^ whilst obesity may result in endothelial dysfunction via with metabolic abnormalities such as free fatty acids, insulin resistance, and chronic inflammation.^47^ Severity of CSVD is associated with both reduced NVC^20^ and with evidence of endothelial dysfunction by blood-brain barrier breakdown^72,73^ and impaired neurovascular responses to non-neuronal stimuli such as carbon dioxide.^16,74,75^ However, these studies included participants with established disease and no previous large study has demonstrated impaired NVC in otherwise healthy brains associated with vascular risk factors.

Our study had several limitations. Firstly, the mean age of the participants in the study was 64, with 95% of subjects younger than 77, limiting the external applicability of these results to the young and elderly. Furthermore, in aiming to determine the association of risk factors in ‘mid-life’ with neurovascular dysfunction before the onset of significant cerebrovascular disease, many of the included population were no longer in mid-life. However, given a similar association with risk factors at inclusion in UKB (ages 40–69) with brain imaging without established disease, this study still supported a role for neurovascular dysfunction prior to established disease. Secondly, MRI imaging was available at a single time point for most participants with limited outcome events after the MRI was performed, such that we could not determine the predictive value of NVC for cerebrovascular events or incident dementia. However, once sufficient repeat MRI scans have been performed and more participants have developed cerebrovascular events or dementia, we will be able to formally test the potential of NVC as a mediator. Thirdly, the task-MRI and derived indices available in UKB were not optimized for measurement of NVC.^76^ Therefore, despite the robust relationships demonstrated, a more precise measure of NVC could be derived both from available UKB data and in future studies with dedicated imaging protocols. Fourthly, the blood biochemistry data was not concurrent to NVC measurements because of UKB’s protocol. Fifthly, diagnoses of hypertension or diabetes were self-reported in UKB rather than diagnosed face-to-face by medical personnel. However, this is likely to have underestimated their incidence, tending to reduce the strength of association. Finally, UKB IDPs currently does not include other CSVD markers, including cerebral microbleeds, enlarged perivascular space, or lacunes. Our study also had a number of strengths. It is the largest ever study of the relationship between vascular risk factors and NVC; it was performed in a largely healthy population, without evidence of significant cerebrovascular disease thus reducing the risk of reverse causation; the NVC response was predominantly dependent on visual cortex, which is likely to be robust to effects of neuronal injury; the magnitude of the effect was large, consistent with a potentially causative mechanism of disease.

Future studies are required to confirm that impaired NVC is present in participants without established disease in independent cohorts, and that it predicts the future development and progression of CSVD and cerebrovascular events. Since age-related cognitive impairments are associated with mid-life vascular risk factors,^77,78^ the potential role of NVC in mediating or contributing to cognitive decline also requires further investigation in longitudinal studies. Furthermore, the use of NVC as a marker of disease activity and treatment target will require optimization of MRI sequences, experimental stimuli and NVC parameters. Finally, the causative role of NVC and its potential as a treatment target can only be proven through interventional studies, including clinical trials, using drugs with proven haemodynamic effects on related targets, such as sildenafil,^16^ cilostazol, or ISMN,^15^ thus leading to clinical trials with longer term clinical outcomes.

In summary, our study demonstrates impaired neurovascular function in individuals with a higher burden of vascular risk factors, even in the absence of clinically apparent cerebrovascular disease. This was consistent for hypertension, diabetes, smoking, and central obesity and confirms its early impact on neurovascular function, its potential causative role in mediating the relationship between risk factor exposure and clinical outcomes, and its potential as a readily-applicable marker in large cohorts and future trials.

## Supplemental Material

sj-pdf-1-jcb-10.1177_0271678X241302172 - Supplemental material for Reduced neurovascular coupling is associated with increased cardiovascular risk without established cerebrovascular disease: A cross-sectional analysis in UK biobank Supplemental material, sj-pdf-1-jcb-10.1177_0271678X241302172 for Reduced neurovascular coupling is associated with increased cardiovascular risk without established cerebrovascular disease: A cross-sectional analysis in UK biobank by Sheng Yang and Alastair John Stewart Webb in Journal of Cerebral Blood Flow & Metabolism
