# Supplementary material for: Reduced neurovascular coupling is associated with increased cardiovascular risk without established cerebrovascular disease: A cross-sectional analysis in UK Biobank
Source: J Cereb Blood Flow Metab. 2024 Nov 22;45(5):897–907. doi: 10.1177/0271678X241302172 (PMC11585009; doi:10.1177/0271678X241302172)
Supplement: sj-pdf-1-jcb-10.1177_0271678X241302172 - Supplemental material for Reduced neurovascular coupling is associated with increased cardiovascular risk without established cerebrovascular disease: A cross-sectional analysis in UK biobank [file sj-pdf-1-jcb-10.1177_0271678X241302172.pdf]

Supplementary Materials

Table of Contents

Table S1. UK Biobank MRI image acquisition protocols and generation pipeline of selected image-derived phenotypes. *2*

Table S2. UK Biobank data included in this study...................................... *4*

Figure S1. Group-defined mask... *6*

Figure S2. Flowchart for subjects' selection. *7*

Figure S3. Normality test. *8*

Table S3. Demographics of subjects with available ASCVD data. *9*

Figure S4. Neurovascular coupling and 10-year cardiovascular disease risk. *10*

Sensitivity Analysis 1...*11*

Table S4. Demographics omitting subjects with lower CSVD burden. 11

Table S5. Association of concurrent age, sex, and vascular risk factors with neurovascular coupling in subjects with lower CSVD burden. 12

Sensitivity Analysis 2...*13*

Table S6. Demographics omitting subjects with higher head motion. 13

Table S7. Association of concurrent age, sex, and vascular risk factors with neurovascular coupling in subjects with lower head motion. 14

Sensitivity Analysis 3...*15*

Table S8. Demographics of subjects with normal blood pressure, defined as SBP <120 and DBP <80. 15

Table S9. Association of concurrent age, sex, and vascular risk factors with neurovascular coupling in subjects with normal blood pressure.................. 16

Table S10. Association of age, sex, and vascular risk factors at baseline with current neurovascular coupling....*17*

Table S11. Association of concurrent age, sex, MAP, PP, and vascular risk factors with neurovascular coupling...*18*

Figure S5. Association between NVC and diabetes. *19*

Figure S6. Blood biochemistry *20*

Table S1. UK Biobank MRI image acquisition protocols and generation pipeline of selected image-derived phenotypes.

The full details of UKB brain MRI acquisition methods can be found in the primary brain imaging documentation: http://biobank.ctsu.ox.ac.uk/crystal/crystal/docs/brain_mri.pdf. The UKB obtained brain MRI data using a Siemens Skyra 3 Tesla scanner, and the parameters for the sequence have been previously published.[1] The UKB brain imaging team utilized FMRIB Software Library (FSL) tools (http://www.fmrib.ox.ac.uk/fsl) to pre-process and analyse T1-weighted 3D magnetization-prepared 180 degrees radiofrequency pulses, rapid gradient-echo, and fMRI data. The analysis pipeline and quality assessment have been previously outlined,[2] wherein the T1-weighted images underwent skull-stripping and bias field correction with the Brain Extraction Tool,[3] followed by segmentation into white and grey matter and cerebrospinal fluid using FMRIB's Automated Segmentation Tool (FAST).[4] Image-derived phenotypes (IDPs) representing objective quantifications of different aspects of brain structure and function were generated, and the pipeline was previously published.[2]

Modality	Description	
T1-weighted structural imaging	Acquisition Protocols	• Resolution: 1x1x1 mm<br>• Field-of-view: 208x256x256 matrix<br>• Duration: 5 minutes<br>• 3D MPRAGE, sagittal, in-plane acceleration iPAT=2, prescan-normalise
	IDPs	• Volume of brain, grey + white matter (normalized for head size) (**Data-field #25009**)
T2-weighted FLAIR structural imaging	Acquisition Protocols	• Resolution: 1.05x1x1 mm<br>• Field-of-view: 192x256x256 matrix<br>• Duration: 6 minutes<br>• 3D SPACE, sagittal, in-plane acceleration iPAT=2, partial Fourier=7/8, fat saturation, elliptical k-space scanning, prescan-normalise
	IDPs	• Total volume of white matter hyperintensities[5] (from T1 and T2_FLAIR images) (**Data-field #25781**) calculated with BIANCA (Brain Intensity AbNormality Classification Algorithm).[6] The total volume of white matter hyperintensities was divided by brain volume to get normalized white matter hyperintensities (nWMH).
	Acquisition	• Resolution: 2.4x2.4x2.4 mm

Task-related fMRI	Protocols	• Field-of-view: 88x88x64 matrix<br>• Duration: 4 minutes (332 timepoints)<br>• TR: 0.735 s, TE: 39 ms<br>• GE-EPI with x8 multi-slice acceleration, no iPAT, flip angle 52°, fat saturation<br>• The task is the Hariri faces/shapes "emotion" task,[7] as implemented in the Human Connectome Project (HCP), but with shorter overall duration and hence fewer total stimulus block repeats. The participants are presented with blocks of trials and asked to decide either which of two faces presented on the bottom of the screen match the face at the top of the screen, or which of two shapes presented at the bottom of the screen match the shape at the top of the screen. The faces have either angry or fearful expressions. The ePrime script that controls the video presented to the participant is derived from the one used by the HCP, and is available at http://biobank.ctsu.ox.ac.uk/crystal/refer.cgi?id=1462.
	IDPs	• Task-related functional brain images (NIFTI) (**Data-field #20249**): Used to identify participants who has undergone fMRI and had available images.<br>• Median z-statistic (in group-defined mask) for shapes activation (Data-field #25042): This is the primary outcome of this study, neurovascular coupling. Pre-processing and task-induced activation modelling was carried out using FEAT (FMRI Expert Analysis Tool).[8] The Featquery tool was used to extract summary statistics for these 4 contrast/mask combinations, for both activation effect size (expressed as a % signal change relative to the overall-image-mean baseline level) and statistical effect size (z-statistic), with each of these summarized across the relevant region-of-interest (ROI) in two ways - median across ROI voxels and 90th percentile across ROI voxels.<br>• Mean tfMRI head motion, averaged across space and time points (**Data-field #25742**): This was estimated from FSL's FEAT and Eddy[8, 9] and estimated the mean, median and 90th percentile over time of the absolute and relative motion (averaged across space) in the task-fMRI.

Table S2. UK Biobank data included in this study.

	Data-fields	Description
**Image-derived phenotypes**		
Structural MRI	Acquisition protocols	Summarized in **Table S1**
	25009	Volume of brain, grey + white matter (normalized for head size)
	25781	Total volume of white matter hyperintensities (from T1 and T2_FLAIR images)
Functional MRI	Acquisition protocols	Summarized in **Table S1**
	20249	Task-related functional brain images (NIFTI)
	25042	Median z-statistic (in group-defined mask) for shapes activation
	25742	Mean tfMRI head motion, averaged across space and time points
**Vascular risk factors**		
Unmodifiable factors	21003	Age
	31	Sex
	21000	Ethnic background
Body size measures	Procedure for body composition measurement	https://biobank.ctsu.ox.ac.uk/crystal/ukb/docs/body_composition.pdf
	21001	Body mass index
	48	Waist circumference
	49	Hip circumference
Blood pressure measures	Procedure for blood pressure measurement	https://biobank.ctsu.ox.ac.uk/crystal/ukb/docs/Bloodpressure.pdf
	4080	Systolic blood pressure, automated reading
	93	Systolic blood pressure, manual reading
	12674	Systolic brachial blood pressure during pulse wave analysis
	4081	Diastolic blood pressure, automated reading
	94	Diastolic blood pressure, manual reading
	12675	Diastolic brachial blood pressure during pulse wave analysis
	2966	Hypertension (self-reported)

Disease measures	2443	Diabetes (self-reported)
	6117	Medication for cholesterol, blood pressure or diabetes
	41270	Diagnoses – ICD10
	41271	Diagnoses – ICD9
Medication	6177	Medication for cholesterol, blood pressure or diabetes
Smoking measures	20116	Smoking status
	20161	Pack years of smoking
	20160	Ever smoked
Blood biochemistry	Biochemistry assay quality procedures	https://biobank.ctsu.ox.ac.uk/crystal/ukb/docs/biomarker_issues.pdf
	30740	Glucose
	30750	Glycated haemoglobin (HbA1c)
	30690	Total cholesterol
	30760	HDL cholesterol
	30780	LDL direct
	30870	Triglycerides

Figure S1. Group-defined mask.

Task-related fMRI group averaged defined mask. Retrieved from: https://biobank.ctsu.ox.ac.uk/showcase/refer.cgi?id=9028.

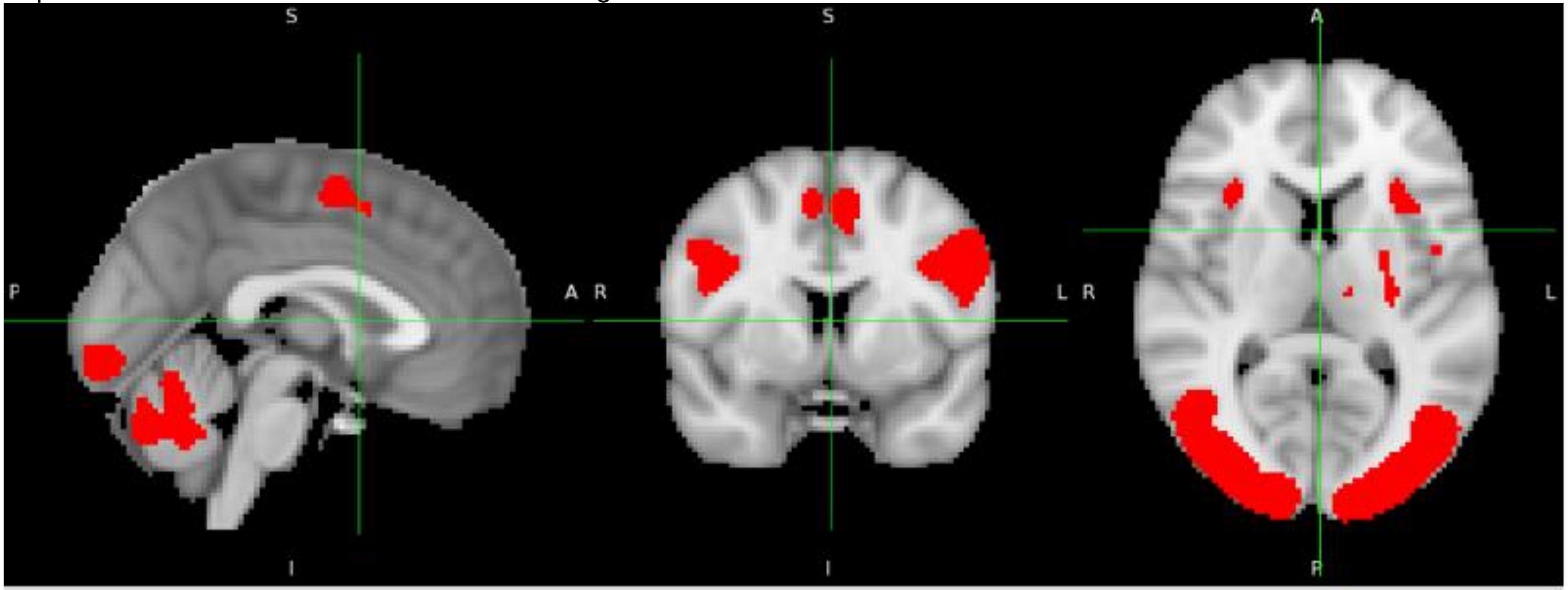

Figure S2. Flowchart for subjects' selection.

CSVD: cerebral small vessel disease; tfMRI: task-related functional magnetic resonance imaging.

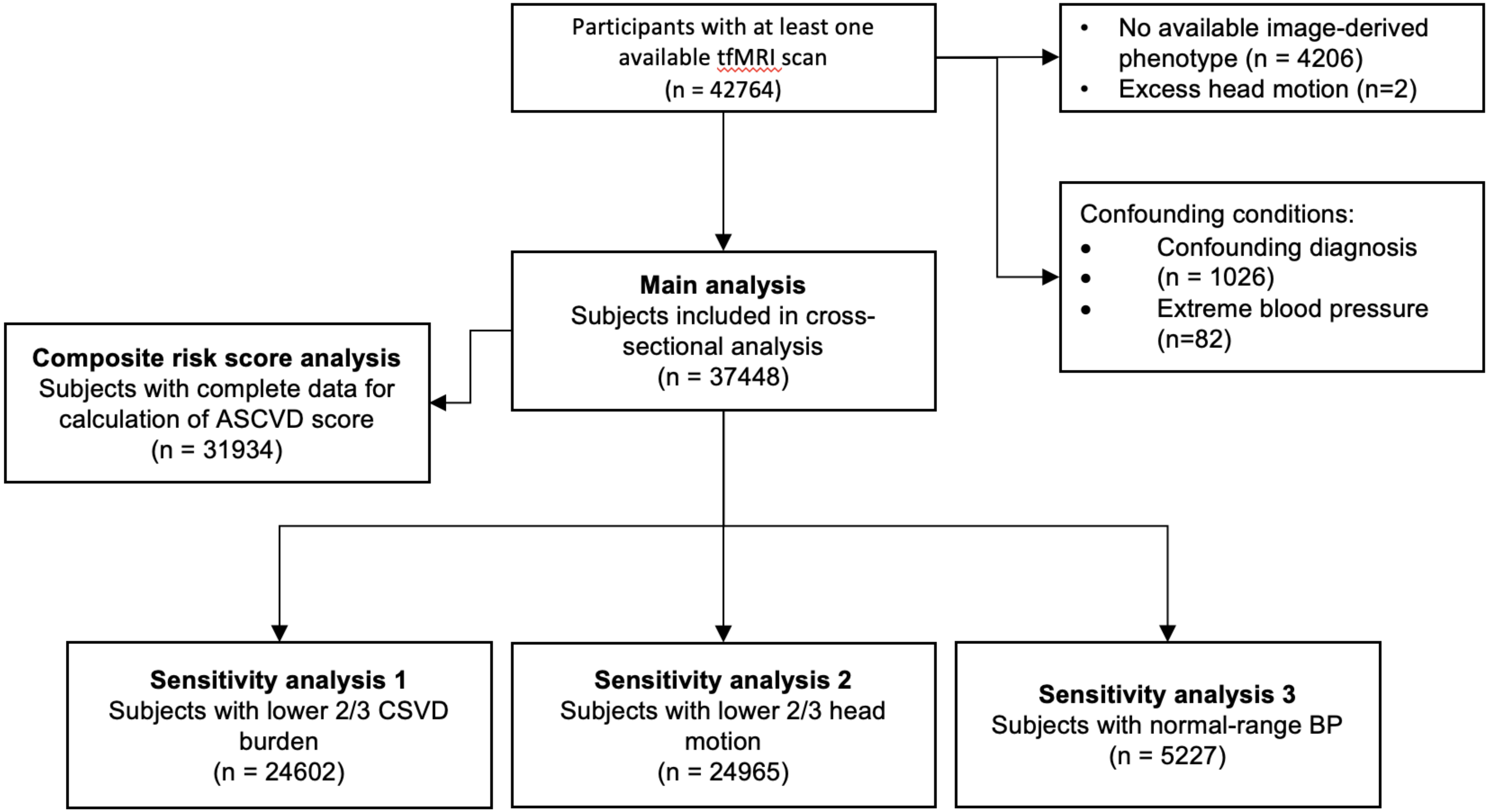

Figure S3. Normality test.

MAP: mean arterial pressure; PP: pulse pressure; WHR: waist-hip ratio.

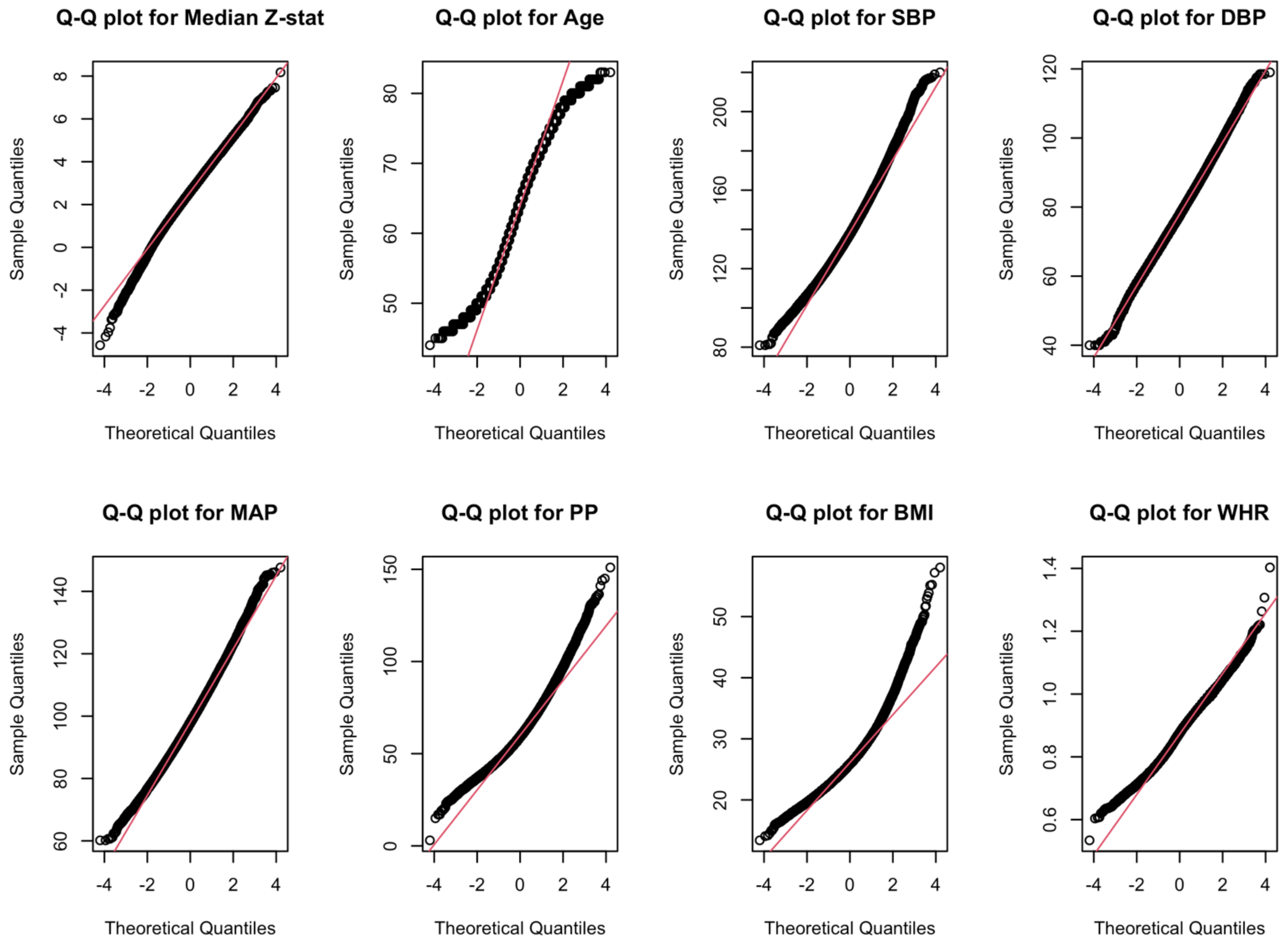

Table S3. Demographics of subjects with available ASCVD data.

Values at the time of fMRI are presented, aside for baseline blood biochemistry measures which was taken on average 9.2 ± 2.1 years before fMRI. Continuous variables given as mean (SD) and frequencies as n (%). NVC: neurovascular coupling; SBP: systolic blood pressure; DBP: diastolic blood pressure; BMI: body mass index; WHR: waist-hip ratio; HbA1c: haemoglobin A1C; HDL: high-density lipoprotein; LDL: low-density lipoprotein.

Variable	Available ASCVD	Whole Population
Participants (n)	31934	37448
NVC (z-statistic)	2.6 (1.4)	2.6 (1.4)
Age	64.0 (7.7)	64.0 (7.7)
Male (n)	15069 (47.2)	17486 (46.7)
White	29150 (91.3)	34159 (91.2)
SBP (mmHg)	139.2 (18.6)	139.2 (18.6)
DBP (mmHg)	78.0 (10.4)	78.0 (10.4)
Weight (kg)	75.9 (15.0)	75.9 (15.0)
Height (cm)	169.0 (9.2)	169.0 (9.2)
BMI (kg/m^2)	26.5 (4.4)	26.5 (4.4)
WHR	0.9 (0.1)	0.9 (0.1)
Current smoker	1948 (6.1)	2279 (6.1)
Ex-smoker	10471 (32.8)	12293 (32.8)
Pack-year [Δ]	5.1 (11.5)	5.0 (11.5)
Hypertension	9630 (30.2)	11206 (29.9)
Diabetes	1919 (6.0)	2254 (6.0)
Hypercholesterolaemia	3134 (9.8)	3696 (9.9)
HbA1c (%) [†]	5.3 (2.6)	5.3 (2.6)
Total cholesterol (mg/dL) [†]	221.6 (41.6)	221.7 (41.7)
Triglycerides (mg/dL) [†]	145.0 (84.4)	145.0 (84.59)
HDL (mg/dL) [†]	57.2 (14.5)	57.2 (14.5)
LDL (mg/dL) [†]	134.8 (31.8)	138.6 (31.9)

[Δ]86.4% (available ASCVD) and 86.2% (whole population) of participants had available smoking pack-year data.

[†]85.3% (available ASCVD) and 85.2% (whole population) of participants had available blood biochemistry data.

Figure S4. Neurovascular coupling and 10-year cardiovascular disease risk.

(a,b) Linear regression representing relationship between NVC and 10-year cardiovascular event risk scores, further stratified by age below or above 65. Area in grey represented 95% CI of NVC of all ages. (c-e) NVC is presented as dots (mean, expressed as z-statistic) with error bars (95% CI). The size of each dot is proportional to the logarithm of the subgroup size. The horizontal dashed line marks the overall mean NVC. ASCVD: atherosclerotic cardiovascular disease; NVC: neurovascular coupling.

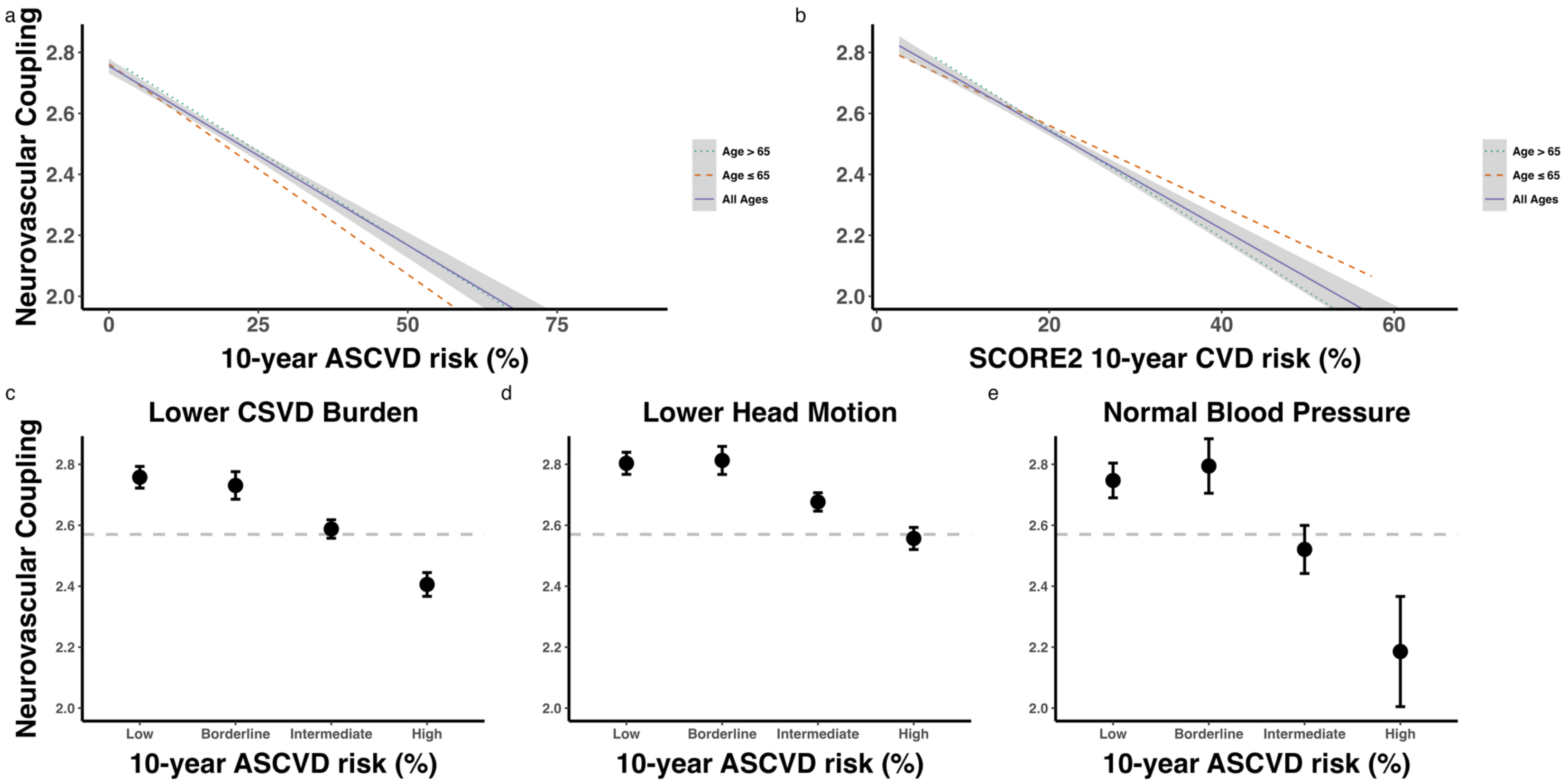

Sensitivity Analysis 1

Table S4. Demographics omitting subjects with lower CSVD burden.

Values at the time of fMRI are presented, aside for baseline blood biochemistry measures which was taken on average 9.1 ± 2.1 years before fMRI. Continuous variables given as mean (SD) and frequencies as n (%). NVC: neurovascular coupling; SBP: systolic blood pressure; DBP: diastolic blood pressure; BMI: body mass index; WHR: waist-hip ratio; HbA1c: haemoglobin A1C; HDL: high-density lipoprotein; LDL: low-density lipoprotein.

**Variable**	**Values**
Participants (n)	24602
NVC (z-statistic)	2.62 (1.4)
Age	61.6 (7.2)
Male (n)	11259 (45.8)
White	22275 (90.5)
SBP (mmHg)	136.1 (17.7)
DBP (mmHg)	77.6 (10.4)
Weight (kg)	75.6 (10.3)
Height (cm)	169.3 (9.2)
BMI (kg/m^2)	26.3 (4.3)
WHR	0.9 (0.1)
Current smoker	1465 (6.0)
Ex-smoker	7462 (30.3)
Pack-year $^{\Delta}$	4.1 (10.0)
Hypertension	5830 (23.7)
Diabetes	1141 (4.6)
Hypercholesterolaemia	1847 (7.5)
HbA1c (%) †	5.3 (2.6)
Total cholesterol (mg/dL) †	220.3 (40.6)
Triglycerides (mg/dL) †	141.54 (84.6)
HDL (mg/dL) †	57.2 (14.3)
LDL (mg/dL) †	137.6 (31.3)

$^{\Delta}$86.4% of participants had available smoking pack-year data.

†85.4% of participants had available blood biochemistry data.

Table S5. Association of concurrent age, sex, and vascular risk factors with neurovascular coupling in subjects with lower CSVD burden.

Associations are presented as standardized coefficients (β) and p-values from generalized linear models. The fully-adjusted model included: age, age^2, sex, SBP, DBP, BMI, smoking, diabetes, hypercholesterolemia. ^Δ^ BMI excluded. † SBP and DBP excluded. Significance level: * p <0.05, ** p <0.01, *** p <0.001. SBP: systolic blood pressure; DBP: diastolic blood pressure; BMI: body mass index; WHR: waist-hip ratio.

Variable	Unadjusted		Age and sex adjusted		Fully adjusted	
	β	p-value	β	p-value	β	p-value
**Age**	-0.052	<0.001***	-	-	-0.043	<0.001***
**Age^2**	-0.015	0.013	-	-	-0.033	<0.001***
**Male Sex**	-0.240	<0.001***	-	-	-0.230	<0.001***
**SBP**	-0.033	<0.001***	0.000	0.96	-0.011	0.24
**DBP**	-0.003	0.60	0.019	0.004**	0.022	0.008**
**BMI**	-0.011	0.08	0.000	0.98	-0.001	0.87
**WHR** Δ	-0.100	<0.001***	-0.037	<0.001***	-0.030	0.001**
**Ex-smoker**	-0.056	<0.001***	-0.037	0.006**	-0.037	0.007**
**Current smoker**	0.049	0.16	0.054	0.12	0.056	0.11
**Hypertension** †	-0.081	<0.001***	-0.030	0.046*	-0.031	0.05
**Diabetes**	-0.100	0.001**	-0.059	0.047*	-0.053	0.08
**Hypercholesterolaemia**	-0.072	0.003**	-0.003	0.89	0.001	0.98

Sensitivity Analysis 2

Table S6. Demographics omitting subjects with higher head motion.

Values at the time of fMRI are presented, aside for baseline blood biochemistry measures which was taken on average 9.0 ± 2.1 years before fMRI. Continuous variables given as mean (SD) and frequencies as n (%). NVC: neurovascular coupling; SBP: systolic blood pressure; DBP: diastolic blood pressure; BMI: body mass index; WHR: waist-hip ratio; HbA1c: haemoglobin A1C; HDL: high-density lipoprotein; LDL: low-density lipoprotein.

**Variable**	**Values**
Participants (n)	24602
NVC (z-statistic)	2.7 (1.3)
Age	62.7 (7.6)
Male (n)	11160 (44.7)
White	22713 (91.0)
SBP (mmHg)	136.6 (18.3)
DBP (mmHg)	77.0 (10.2)
Weight (kg)	72.5 (13.0)
Height (cm)	169.2 (9.2)
BMI (kg/m^2)	25.2 (3.5)
WHR	0.9 (0.1)
Current smoker	1351 (5.4)
Ex-smoker	7582 (30.4)
Pack-year [Δ]	3.9 (9.4)
Hypertension	5889 (23.6)
Diabetes	899 (3.6)
Hypercholesterolaemia	1902 (7.6)
HbA1c (%) [†]	5.3 (2.6)
Total cholesterol (mg/dL) [†]	221.3 (40.8)
Triglycerides (mg/dL) [†]	133.7 (77.6)
HDL (mg/dL) [†]	58.9 (14.7)
LDL (mg/dL) [†]	137.6 (31.3)

[Δ]86.5% of participants had available smoking pack-year data.

[†]85.2% of participants had available blood biochemistry data.

Table S7. Association of concurrent age, sex, and vascular risk factors with neurovascular coupling in subjects with lower head motion.

Associations are presented as standardized coefficients (β) and p-values from generalized linear models. The fully-adjusted model included: age, age^2, sex, SBP, DBP, BMI, smoking, diabetes, hypercholesterolemia. ^Δ BMI excluded. † SBP and DBP excluded. Significance level: * p <0.05, ** p <0.01, *** p <0.001. SBP: systolic blood pressure; DBP: diastolic blood pressure; BMI: body mass index; WHR: waist-hip ratio.

Variable	Unadjusted		Age and sex adjusted		Fully adjusted	
	β	p-value	β	p-value	β	p-value
**Age**	-0.020	0.001**	-	-	-0.007	0.38
**Age^2**	-0.026	<0.001***	-	-	-0.026	<0.001***
**Male Sex**	-0.250	<0.001***	-	-	-0.250	<0.001***
**SBP**	-0.015	0.015*	0.009	0.18	-0.004	0.64
**DBP**	0.000	0.95	0.024	<0.001***	0.021	0.013*
**BMI**	-0.004	0.61	0.018	0.015*	0.015	0.048*
**WHR** ^Δ	-0.094	<0.001***	-0.011	0.24	-0.005	0.58
**Ex-smoker**	-0.047	0.001**	-0.035	0.009**	-0.037	0.006**
**Current smoker**	0.015	0.67	0.028	0.44	0.033	0.36
**Hypertension** †	-0.048	0.001**	-0.008	0.61	-0.012	0.43
**Diabetes**	-0.100	0.002**	-0.054	0.10	-0.057	0.087
**Hypercholesterolaemia**	-0.056	0.016*	-0.005	0.84	-0.005	0.84

Sensitivity Analysis 3

Table S8. Demographics of subjects with normal blood pressure, defined as SBP <120 and DBP <80.

Values at the time of fMRI are presented, aside for baseline blood biochemistry measures which was taken on average 9.0 ± 2.0 years before fMRI. Continuous variables given as mean (SD) and frequencies as n (%). NVC: neurovascular coupling; SBP: systolic blood pressure; DBP: diastolic blood pressure; BMI: body mass index; WHR: waist-hip ratio; HbA1c: haemoglobin A1C; HDL: high-density lipoprotein; LDL: low-density lipoprotein.

**Variable**	**Values**
Participants (n)	5227
NVC (z-statistic)	2.7 (1.4)
Age	60.2 (7.2)
Male (n)	1395 (26.7)
White	4653 (89.0)
SBP (mmHg)	112.2 (6.1)
DBP (mmHg)	67.4 (6.5)
Weight (kg)	69.1 (12.5)
Height (cm)	167.3 (8.6)
BMI (kg/m^2)	24.6 (3.8)
WHR	0.8 (0.1)
Current smoker	355 (6.8)
Ex-smoker	1530 (29.3)
Pack-year $^{\Delta}$	4.0 (9.9)
Hypertension	513 (9.8)
Diabetes	160 (3.1)
Hypercholesterolaemia	311 (5.9)
HbA1c (%) †	5.3 (2.5)
Total cholesterol (mg/dL) †	216.0 (40.0)
Triglycerides (mg/dL) †	118.7 (69.7)
HDL (mg/dL) †	60.6 (14.5)
LDL (mg/dL) †	132.6 (30.7)

$^{\Delta}$86.1% of participants had available smoking pack-year data.

†84.6% of participants had available blood biochemistry data.

Table S9. Association of concurrent age, sex, and vascular risk factors with neurovascular coupling in subjects with normal blood pressure.

Associations are presented as standardized coefficients (β) and p-values from generalized linear models. The fully-adjusted model included: age, age^2, sex, SBP, DBP, BMI, smoking, diabetes, hypercholesterolemia. $^{\Delta}$BMI excluded. † SBP and DBP excluded. Significance level: * p <0.05, ** p <0.01, *** p <0.001. SBP: systolic blood pressure; DBP: diastolic blood pressure; BMI: body mass index; WHR: waist-hip ratio.

Variable	Unadjusted		Age and sex adjusted		Fully adjusted	
	β	p-value	β	p-value	β	p-value
**Age**	-0.049	0.001**	-	-	-0.051	0.006**
**Age^2**	-0.017	<0.001***	-	-	-0.046	0.001**
**Male Sex**	-0.250	<0.001***	-	-	-0.240	<0.001***
**SBP**	0.026	0.54	0.087	0.042*	0.050	0.29
**DBP**	0.063	0.005**	0.066	0.003**	0.038	0.13
**BMI**	-0.004	0.81	0.012	0.43	0.008	0.62
**WHR** $^{\Delta}$	-0.094	<0.001***	-0.028	0.14	-0.020	0.30
**Ex-smoker**	-0.031	0.30	-0.025	0.42	-0.026	0.40
**Current smoker**	0.009	0.89	0.011	0.88	0.015	0.82
**Hypertension** †	-0.110	0.020*	-0.040	0.41	-0.028	0.59
**Diabetes**	-0.150	0.07	-0.096	0.23	-0.067	0.41
**Hypercholesterolaemia**	-0.160	0.008**	-0.075	0.21	-0.067	0.27

Table S10. Association of age, sex, and vascular risk factors at baseline with current neurovascular coupling.

Associations are presented as standardized coefficients (β) and p-values from generalized linear models. The fully-adjusted model included: age at baseline, (age at baseline)^2, sex, SBP at baseline, DBP at baseline, BMI at baseline, smoking status at baseline, diabetes at baseline, hypercholesterolemia at baseline, and time difference between baseline data collection and fMRI. $^{\Delta}$ BMI excluded. † SBP and DBP excluded. Significance level: * p <0.05, ** p <0.01, *** p <0.001. SBP: systolic blood pressure; DBP: diastolic blood pressure; BMI: body mass index; WHR: waist-hip ratio.

**Variable**	**Unadjusted**		**Age and sex adjusted**		**Fully adjusted**	
	β	p-value	β	p-value	β	p-value
**Age**	-0.055	<0.001***	-	-	-0.025	0.09
**Age^2**	-0.034	<0.001***	-	-	-0.033	0.012*
**Male Sex**	-0.250	<0.001***	-	-	-0.170	<0.001***
**SBP**	-0.038	<0.001***	0.007	0.22	-0.046	0.027*
**DBP**	-0.020	<0.001***	0.011	0.045*	0.018	0.39
**BMI**	-0.030	0.020*	-0.014	0.28	-0.007	0.60
**WHR** $^{\Delta}$	-0.100	<0.001***	-0.019	0.006*	-0.020	0.007**
**Ex-smoker**	-0.074	<0.001***	-0.042	<0.001***	-0.044	0.120
**Current smoker**	-0.100	<0.001***	-0.087	<0.001***	-0.110	0.07
**Hypertension** †	-0.110	<0.001***	-0.048	<0.001***	-0.059	<0.001***
**Diabetes**	-0.130	<0.001***	-0.079	<0.001***	-0.018	0.75
**Hypercholesterolaemia**	-0.870	<0.001***	-0.009	0.60	0.004	0.92

Table S11. Association of concurrent age, sex, MAP, PP, and vascular risk factors with neurovascular coupling.

Associations are presented as standardized coefficients (β) and p-values from generalized linear models. The fully-adjusted model included: age, age^2, sex, SBP, DBP, BMI, smoking, diabetes, hypercholesterolemia. $^{\Delta}$BMI excluded. † MAP and PP excluded. Significance level: * p <0.05, ** p <0.01, *** p <0.001. MAP: mean arterial pressure; PP: pulse pressure; BMI: body mass index; WHR: waist-hip ratio.

Variable	Unadjusted		Age and sex adjusted		Fully adjusted	
	β	p-value	β	p-value	β	p-value
**Age**	-0.072	<0.001***	-	-	-0.056	<0.001***
**Age^2**	-0.038	<0.001***	-	-	-0.039	<0.001***
**Male Sex**	-0.250	<0.001***	-	-	-0.240	<0.001***
**MAP**	-0.023	<0.001***	0.008	0.14	0.014	0.021*
**PP**	-0.045	<0.001***	-0.013	0.027*	-0.012	0.06
**BMI**	-0.015	0.003**	-0.003	0.53	-0.002	0.74
**WHR**	-0.110	<0.001***	-0.038	<0.001***	-0.029	<0.001***
**Ex-smoker**	-0.077	<0.001***	-0.046	<0.001***	-0.047	<0.001***
**Current smoker**	-0.023	0.43	-0.020	0.48	-0.017	0.55
**Hypertension †**	-0.110	<0.001***	-0.048	<0.001***	-0.059	<0.001***
**Diabetes**	-0.130	<0.001***	-0.079	<0.001***	-0.073	0.001**
**Hypercholesterolaemia**	-0.870	<0.001***	-0.009	0.60	0.000	0.99

Figure S5. Association between NVC and diabetes.

The mean neurovascular coupling (z-statistic) is given, with error bars corresponding to 95% confidence interval. The horizontal dashed line marks the overall mean median z-statistic. Between-group comparisons by ANOVA ($p<0.05$ for all variables) with post-hoc t-test (* $p <0.05$, ** $p <0.01$, *** $p<0.001$).

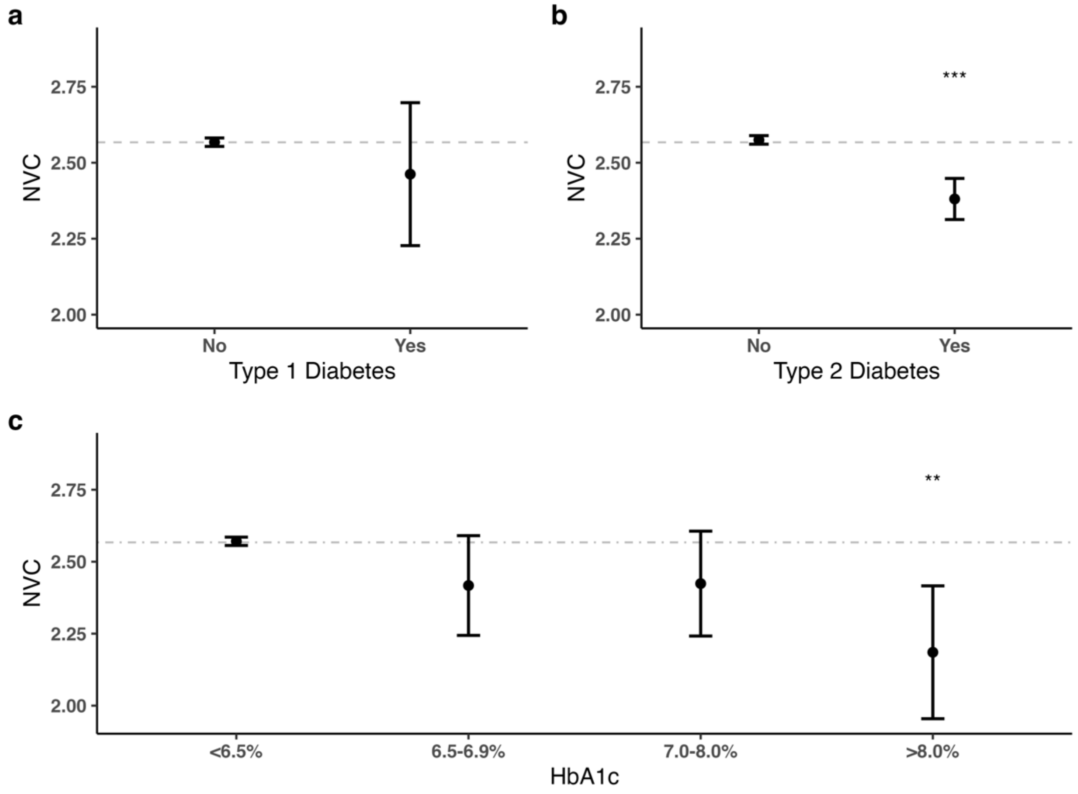

Figure S6. Blood biochemistry

The mean neurovascular coupling (z-statistic) is given, with error bars corresponding to 95% confidence interval. The horizontal dashed line marks the overall mean median z-statistic. Between-group comparisons by ANOVA (p<0.05 for all variables) with post-hoc t-test (* p <0.05, ** p <0.01, *** p<0.001).

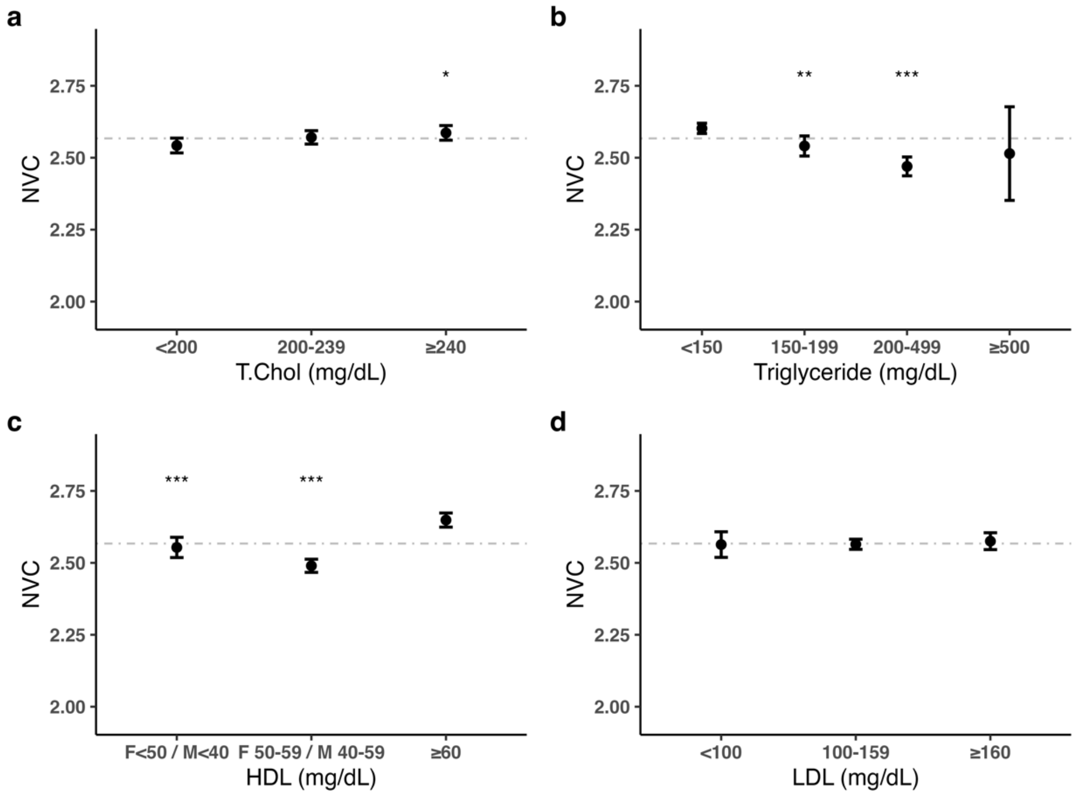

Reference

1. Miller KL, Alfaro-Almagro F, Bangerter NK, et al. Multimodal population brain imaging in the UK Biobank prospective epidemiological study. *Nat Neurosci* 2016; 19: 1523-1536. 20160919. DOI: 10.1038/nn.4393.

2. Alfaro-Almagro F, Jenkinson M, Bangerter NK, et al. Image processing and Quality Control for the first 10,000 brain imaging datasets from UK Biobank. *Neuroimage* 2018; 166: 400-424. 20171024. DOI: 10.1016/j.neuroimage.2017.10.034.

3. M. Jenkinson MP, S. Smith. BET2: MR-based estimation of brain, skull and scalp surfaces. *Eleventh Annual Meeting of the Organization for Human Brain Mapping*. Toronto, Ontario, Canada2005.

4. Zhang Y, Brady M and Smith S. Segmentation of brain MR images through a hidden Markov random field model and the expectation-maximization algorithm. *IEEE Trans Med Imaging* 2001; 20: 45-57. DOI: 10.1109/42.906424.

5. Wardlaw JM, Smith EE, Biessels GJ, et al. Neuroimaging standards for research into small vessel disease and its contribution to ageing and neurodegeneration. *Lancet Neurol* 2013; 12: 822-838. DOI: 10.1016/S1474-4422(13)70124-8.

6. Griffanti L, Zamboni G, Khan A, et al. BIANCA (Brain Intensity AbNormality Classification Algorithm): A new tool for automated segmentation of white matter hyperintensities. *Neuroimage* 2016; 141: 191-205. 20160709. DOI: 10.1016/j.neuroimage.2016.07.018.

7. Hariri AR, Tessitore A, Mattay VS, et al. The amygdala response to emotional stimuli: a comparison of faces and scenes. *Neuroimage* 2002; 17: 317-323. DOI: 10.1006/nimg.2002.1179.

8. Woolrich MW, Ripley BD, Brady M, et al. Temporal autocorrelation in univariate linear modeling of FMRI data. *Neuroimage* 2001; 14: 1370-1386. DOI: 10.1006/nimg.2001.0931.

9. Andersson JLR, Graham MS, Zsoldos E, et al. Incorporating outlier detection and replacement into a non-parametric framework for movement and distortion correction of diffusion MR images. *Neuroimage* 2016; 141: 556-572. 20160705. DOI: 10.1016/j.neuroimage.2016.06.058.
